# Supplementary material for: Exogenous glutathione maintains the postharvest quality of mango fruit by modulating the ascorbate-glutathione cycle
Source: PeerJ. 2023 Aug 22;11:e15902. doi: 10.7717/peerj.15902 (PMC10452625; doi:10.7717/peerj.15902)
Supplement: Supplemental Information 1 [file peerj-11-15902-s001.docx]

| Gene name | Primer sequence (5’-3’) | PCR produce size (bp) |
| --- | --- | --- |
| Actin | F: GCCGTGACCTTACAGATGC | 204 |
|  | R: TGGTGATAACCTGCCCGT | 204 |
| SOD | F: CACTACAAAGCAGCACATTCAC | 195 |
|  | R: GAGTTTGAGAGAGACGCCG | 195 |
| POD | F: ACCAATTCCAACTACACAGAGC | 188 |
|  | R: CCATTCACATCAACCTTATCAA | 188 |
| CAT | F: CATTTTGCCCTGGTATTGTG | 101 |
|  | R: CAGACGGTGCCTTTGAGTAT | 101 |
| APX | F: GGAGTGGTTGGGGAAAATC | 165 |
|  | R: TGACAGCATCGGTAGGCA | 165 |
| MDHAR | F: CTCGTCAATCCGCACAACA | 114 |
|  | R: TCCTGGGGCTCCCTTCAT | 114 |
| DHAR | F: TCACTGTCAACAAATGGCTCT | 206 |
|  | R: ACTTCACTACTGGCACCTTCC | 206 |
| GR | F: TACGAAGAACTTGGGATTGG | 240 |
|  | R: GGTTGGGAAAACACAGCAGAT | 240 |

Table S1.The primer sequences by qRT-PCR analysis.
